# EACVI survey on the use of multi-modality cardiovascular imaging in immune-mediated inflammatory diseases

**DOI:** 10.1093/ehjimp/qyag094

**Published:** 2026-05-16

**Authors:** Marta Peverelli, Beth Whittington, Theo Pezel, Tor Biering-Sørensen, Simona B Botezatu, Robert Manka, Sanjeev Bhattacharyya, José F Rodríguez Palomares, Ahmet Demirkiran, Maria Lembo, Jonathan R Weir-McCall, Timothy Fairbairn, Michelle C Williams, Taryn Youngstein, Ziad Mallat, Declan P O’Regan, Maya H Buch, Vanessa M Ferreira, Sven Plein, Anna Baritussio, Marc R Dweck, Jason M Tarkin

**Affiliations:** Victor Phillip Dahdaleh Heart & Lung Research Institute, University of Cambridge, Papworth Road, Cambridge Biomedical Campus, Cambridge CB2 0BB, UK; British Heart Foundation Centre for Research Excellence, University of Edinburgh, Edinburgh, The Chancellor's Building, Little France Crescent, Midlothian, Edinburgh EH26 0NL, UK; Department of Cardiology, University Hospital of Lariboisiere, Inserm MASCOT—UMRS 942, (Assistance Publique des Hôpitaux de Paris, AP-HP), Université Paris Cité, Paris 75010, France; MIRACL.ai Laboratory, Multimodality Imaging for Research and Analysis Core Laboratory and Artificial Intelligence, University Hospital of Lariboisiere (AP-HP), Paris 75010, France; Gentofte University Hospital, Copenhagen, Denmark; Emergency Institute for Cardiovascular Diseases Prof. Dr. C.C. Iliescu, University of Medicine and Pharmacy Carol Davila, Euroecolab, Bucharest, Romania; Department of Cardiology, University Hospital Zurich, Zurich, Switzerland; St Bartholomew's Hospital, London, UK; Department of Cardiology, Vall d’Hebron Hospital Universitari, Vall d’Hebron Barcelona Hospital Campus, Barcelona, Spain; Cardiovascular Diseases, Vall d′Hebron Institut de Recerca (VHIR), Vall d’Hebron Barcelona Hospital Campus, Barcelona, Spain; Department of Medicine, Universitat Autònoma de Barcelona, Bellaterra, Spain; CIBER de Enfermedades Cardiovasculares, Instituto de Salud Carlos III, Madrid, Spain; University of Health Sciences, Istanbul, Türkiye; Amsterdam UMC, Amsterdam, The Netherlands; Department of Advanced Biomedical Sciences, Federico II University of Naples, Italy; Biomedical Engineering and Imaging Sciences, King's College London, London, UK; Liverpool Centre for Cardiovascular Science, Liverpool Heart and Chest Hospital, Liverpool, UK; British Heart Foundation Centre for Research Excellence, University of Edinburgh, Edinburgh, The Chancellor's Building, Little France Crescent, Midlothian, Edinburgh EH26 0NL, UK; Medical Research Council Laboratory of Medical Sciences, Imperial College London, London, UK; Victor Phillip Dahdaleh Heart & Lung Research Institute, University of Cambridge, Papworth Road, Cambridge Biomedical Campus, Cambridge CB2 0BB, UK; Medical Research Council Laboratory of Medical Sciences, Imperial College London, London, UK; University of Manchester, Manchester, UK; NIHR Manchester Biomedical Research Centre, Manchester, UK; Radcliffe Department of Medicine, University of Oxford, Oxford, UK; Leeds Institute of Cardiovascular and Metabolic Medicine, University of Leeds, Leeds, UK; Department of Cardiac, Thoracic, Vascular Sciences and Public Health, Padua University Hospital, Padua, Italy; British Heart Foundation Centre for Research Excellence, University of Edinburgh, Edinburgh, The Chancellor's Building, Little France Crescent, Midlothian, Edinburgh EH26 0NL, UK; Victor Phillip Dahdaleh Heart & Lung Research Institute, University of Cambridge, Papworth Road, Cambridge Biomedical Campus, Cambridge CB2 0BB, UK

**Keywords:** multi-modality cardiovascular imaging, immune-mediated inflammatory diseases, cardio-rheumatology

## Abstract

**Aims:**

To scope the current European landscape of multi-modality cardiovascular imaging practices in immune-mediated inflammatory diseases (IMIDs).

**Methods and results:**

An electronic (e)-survey was distributed to members of the European Association of Cardiovascular Imaging (EACVI) about imaging in cardio-rheumatology. A parallel e-survey focused on the use of coronary computed tomography (CT) for cardiovascular disease (CVD) risk stratification in IMIDs was conducted via the British Society of Cardiovascular Imaging (BSCI). Of the total 111 respondents to the EACVI survey, 92 (82.9%) were consultant-grade physicians and 68 (61.3%) worked in tertiary centres. These individuals had varied experiences in cardio-rheumatology, with limited access to dedicated cardio-rheumatology services and training opportunities. Sixty-nine (62.2%) used coronary CT to guide preventive therapies in IMID patients with borderline risk scores and cardiac symptoms, with a preference for coronary CT angiography over coronary artery calcium scoring alone in this setting. These findings about coronary CT were corroborated in the parallel survey of 54 BSCI members. When using cardiovascular magnetic resonance imaging (CMR) to detect myocardial involvement in IMIDs, 73/111 (67.6%) perceived a need for IMID-specific CMR criteria beyond the current modified Lake Louise criteria for myocarditis. In terms of nuclear imaging, timely access to ^18^F-fluorodeoxyglucose positron emission tomography imaging was identified as a barrier for diagnosing large-vessel vasculitis, and 61/111 (57%) of responders felt that the development of novel radionuclide tracers should be a future research priority.

**Conclusion:**

This European survey highlights the need for dedicated cardio-rheumatology services and specialist training, and further evidence to inform future clinical practice recommendations on multi-modality cardiovascular imaging in IMIDs.

## Graphical Abstract

**Graphical Abstract:**
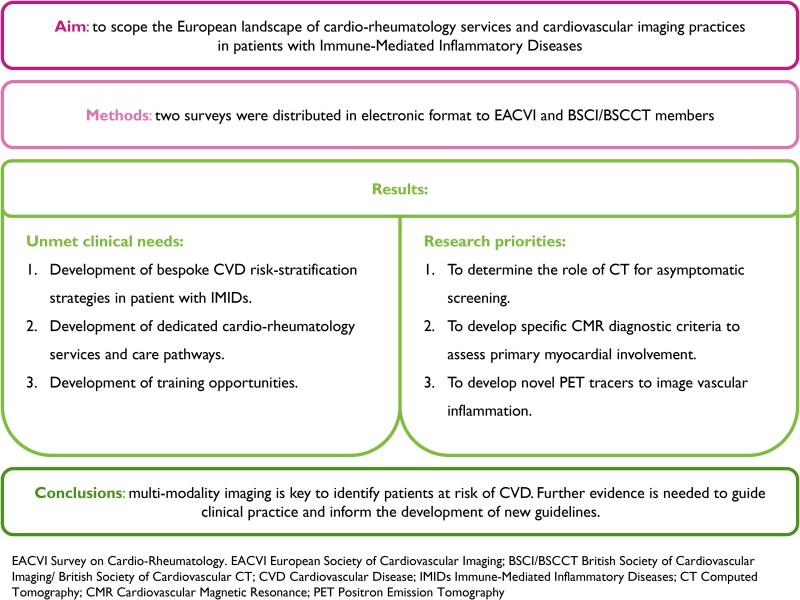

## Introduction

Immune-mediated inflammatory diseases (IMIDs) are a group of heterogeneous clinical conditions associated with systemic inflammation. Rheumatological IMIDs include rheumatoid arthritis, psoriatic arthritis, systemic lupus erythematosus, systemic sclerosis, and vasculitis, among others. It is well established that people with IMIDs are at an increased risk of cardiovascular disease (CVD) compared to the general population, including both atherosclerotic CVD and other cardiovascular complications such as myo-pericardial involvement.

In a population-based analysis of over 22 million individuals in the United Kingdom spanning 19 autoimmune conditions, the risk of incident CVD was significantly elevated in IMIDs, with hazard ratios ranging from 1.4 to 3.6 compared with matched controls.^1^ This excess risk can largely be attributed to systemic inflammation, with likely both shared and distinct inflammatory pathways responsible for cardiovascular and other organ involvement in IMIDs.^2^

Although international clinical practice guidelines acknowledge the increased cardiovascular risk in IMID populations,^3,4^ risk assessment remains frequently underestimated in routine practice and undertreated, resulting in increased morbidity and mortality. QRISK3 is the only commonly used clinical risk assessment tool that also accounts for the presence of an IMID diagnosis. However, its use in this context has not been found to reliably improve the overall accuracy of CVD risk prediction.^5^ The role of multi-modality cardiovascular imaging for CVD diagnosis and guiding management in patients with IMIDs is rapidly evolving; however, real-world practice remains variable across Europe.

This survey aimed to characterize the current European landscape of cardio-rheumatology services and cardiovascular imaging practices in patients with IMIDs.

## Methods

An electronic (e)-survey was conducted by the European Association of Cardiovascular Imaging (EACVI) Scientific Initiatives Committee, with support from the UK Cardio-IMID Partnership. Data were collected in accordance with criteria established by the international EACVI survey network.^6^ The e-survey was released via an online platform on 30 September 2024 and accepted responses until 11 March 2025. Invitations to complete the survey were disseminated via email to all EACVI members.

The survey consisted of 22 questions, including single, multiple-choice, Likert-scale questions, and a clinical scenario addressed to the European cardiovascular imaging community.

In parallel, a more focused survey about the use of coronary computed tomography (CT) for CVD risk stratification in IMIDs was conducted in the United Kingdom via the British Society of Cardiovascular Imaging/British Society of Cardiovascular CT (BSCI/BSCCT), with the support of the British Heart Foundation Clinical Research Collaborative. The latter e-survey was distributed via email to all BSCI members and comprised 20 questions aligned to the EACVI survey, with a pre-specified plan for joint publication. See the Supplementary data online, *methods* for a complete list of the questions included in these two surveys.

To incorporate patient perspectives, a dedicated patient and public involvement and engagement in research session was conducted with the Patient Advisory Group of the UK Cardio-IMID partnership on 20 March 2025.

## Results

**Figure 1:**
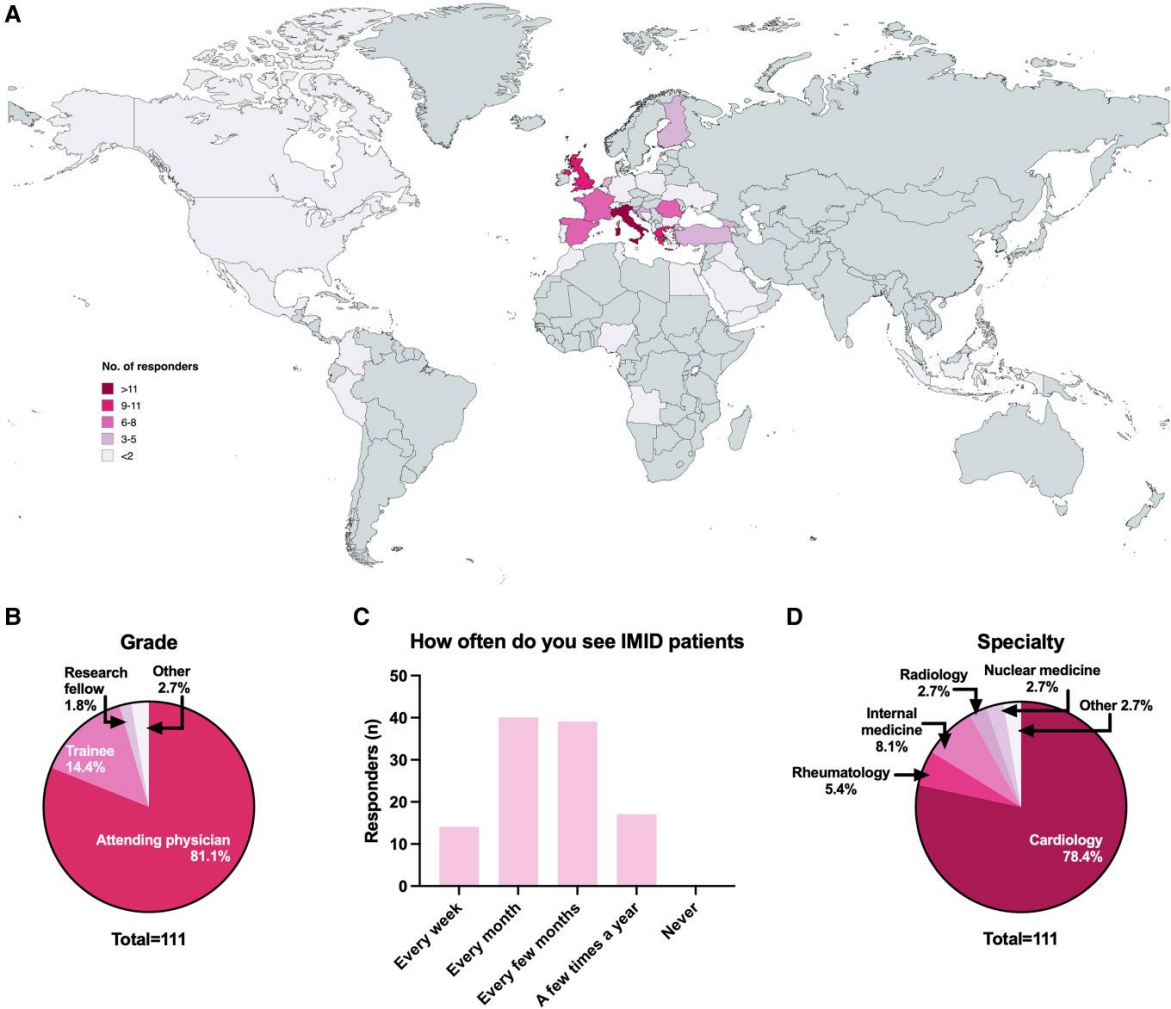
Distribution of participants to the EACVI survey. Graph shows the number of responders per country (*A*), professional role (*B*), cardio-rheumatology experience (*C*), and clinical specialty (*D*).

A total of 111 EACVI members from 39 countries took part in the main survey. Ninety-three responders (83.8%) were from Europe, and 18 (16.2%) were from non-European countries (*Figure 1A*). Most of the participants (*n* = 92, 82.9%) were consultant-grade physicians (*Figure 1B*) with variable experience in the field of cardio-rheumatology (*Figure 1C*), and the majority (*n* = 68, 61.3%) were employed in a tertiary centre. All but one responder were medical doctors (*n* = 110, 99.1%). Reflecting the membership of EACVI, cardiology was the most represented medical specialty in the survey and accounted for 87 (78.4%) of responses, followed by internal medicine (*n* = 9, 8.1%), rheumatology (*n* = 6, 5.4%), radiology (*n* = 3, 2.7%), nuclear medicine (*n* = 3, 2.7%), and other medical specialties (*n* = 3, 2.7%) (*Figure 1D*). A lack of dedicated cardio-rheumatology training opportunities was noted, with as many as 60 participants (54.1%) indicating that training in this field was not available to them locally, and a further 14 (12.6%) who did not know of any such opportunities at all. Of all the imaging modalities, echocardiography was the most readily available and could be accessed by 74 (66.7%) of responders in fewer than 4 weeks.

**Figure 2:**
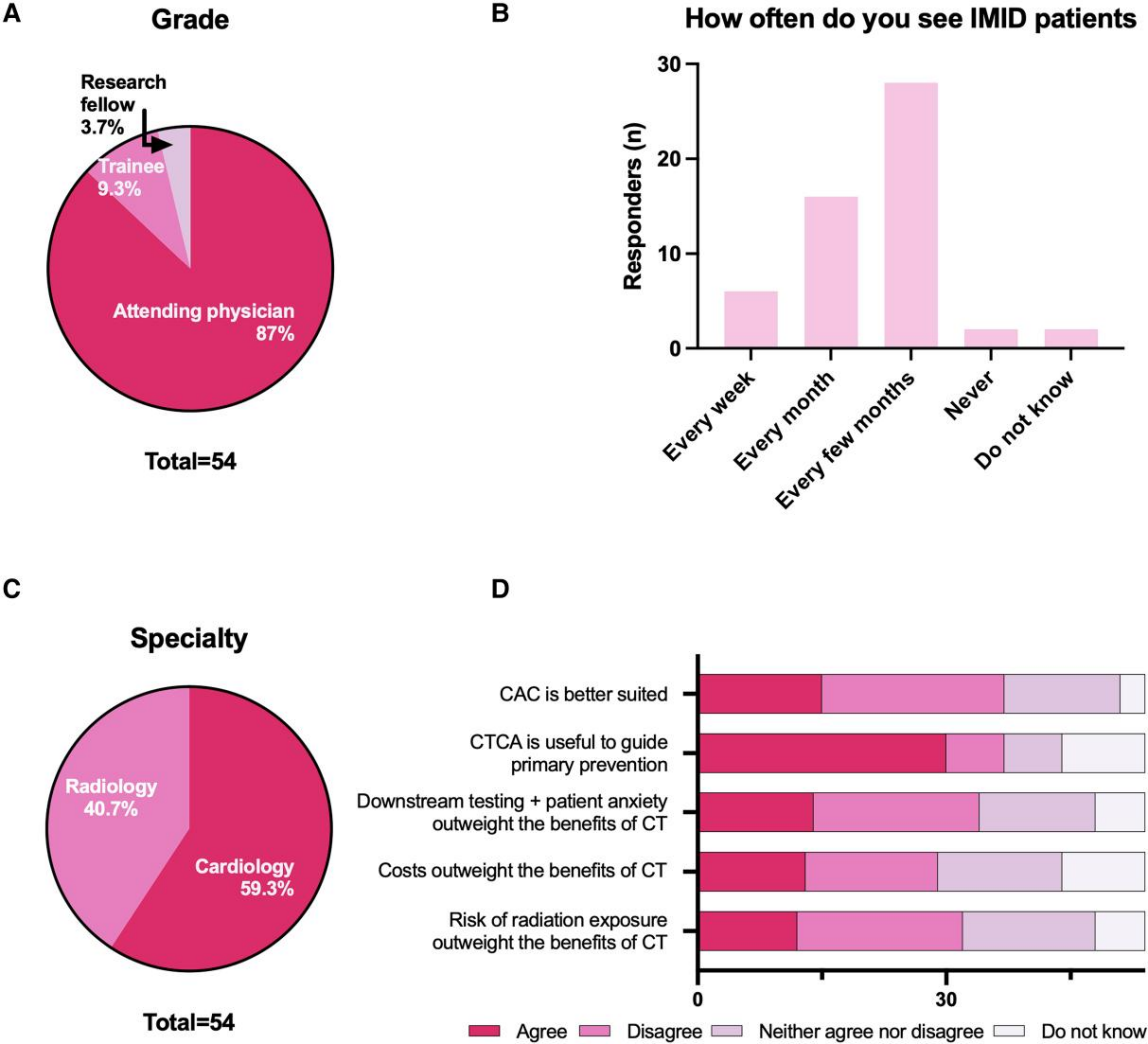
BSCI/BSCCT survey on the use of coronary CT for CVD risk stratification in IMIDs. Graphs show distribution of participants by professional role (*A*), cardio-rheumatology experience (*B*), and clinical specialty (*C*), as well as perceived advantages and disadvantages of CT in the setting of primary prevention (*D*).

Fifty-four people, the majority working in England (*n* = 43, 79.6%), Scotland (*n* = 2, 3.7%), and Wales (*n* = 1, 1.9%) responded to the more focused BSCI/BSCCT survey. Their baseline demographics largely matched those described for the main survey in terms of training grade (*Figure 2A*) and sub-specialty experience (*Figure 2B*). However, a greater number of cardio-thoracic radiologists took part in the survey, accounting for 22 (40.7%) of responses (*Figure 2C*).

### Atherosclerotic CVD risk-stratification in IMIDs

**Figure 3:**
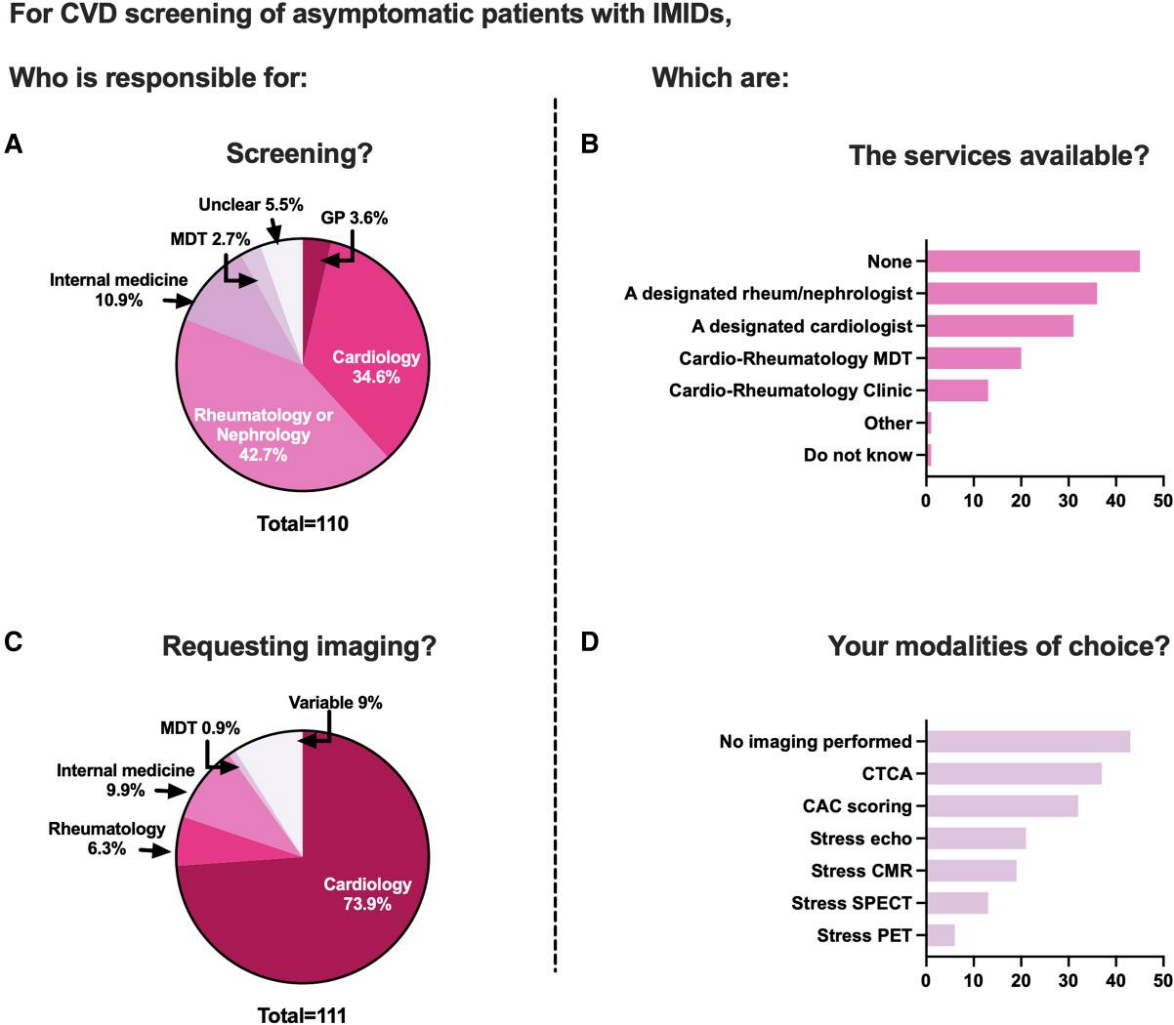
Approach to CVD screening in asymptomatic patients with IMIDs. Graphs showing responses to questions about which clinical specialties are responsible for carrying out CVD screening (*A*) and requesting cardiovascular imaging (*C*) in IMIDs. Summary of cardio-rheumatology services available (*B*) and cardiovascular imaging modalities of choice for asymptomatic CVD screening in IMIDs (*D*). CTCA, coronary CT angiography; GP, general practitioner; MDT, multidisciplinary team; SPECT, single photon emission computed tomography.

It was noted that CVD screening in asymptomatic IMID patients generally occurs in secondary care, with only four individuals (3.6%) stating that this is a primary care responsibility. Reflecting a lack of standardized care pathways, 47 responders (42.7%) indicated that CVD screening was primarily undertaken by either a rheumatologist or a nephrologist, and 38 (34.6%) by a cardiologist (*Figure 3A*). A multi-disciplinary team meeting or specialist cardio-rheumatology clinic was only available in a minority of centres (*Figure 3B*). Ninety-two participants (82.9%) felt that their hospital would benefit from a dedicated cardio-rheumatology service. Eighty-two doctors (73.9%) agreed that the role of requesting and interpretation of cardiovascular imaging investigations was typically performed by cardiologists (*Figure 3C*). While 43 doctors (31 cardiologists and 12 non-cardiologists; 38.7%) would not routinely recommend imaging for atherosclerotic CVD risk-stratification in patients with no cardiac symptoms, 53 (47.8%) use coronary CT in this setting with either coronary artery calcium (CAC) scoring or coronary CT angiography (CCTA), whilst 40 (36%) use stress perfusion imaging (*Figure 3D*).

### Use of coronary CT for CVD risk-stratification in IMIDs

**Figure 4:**
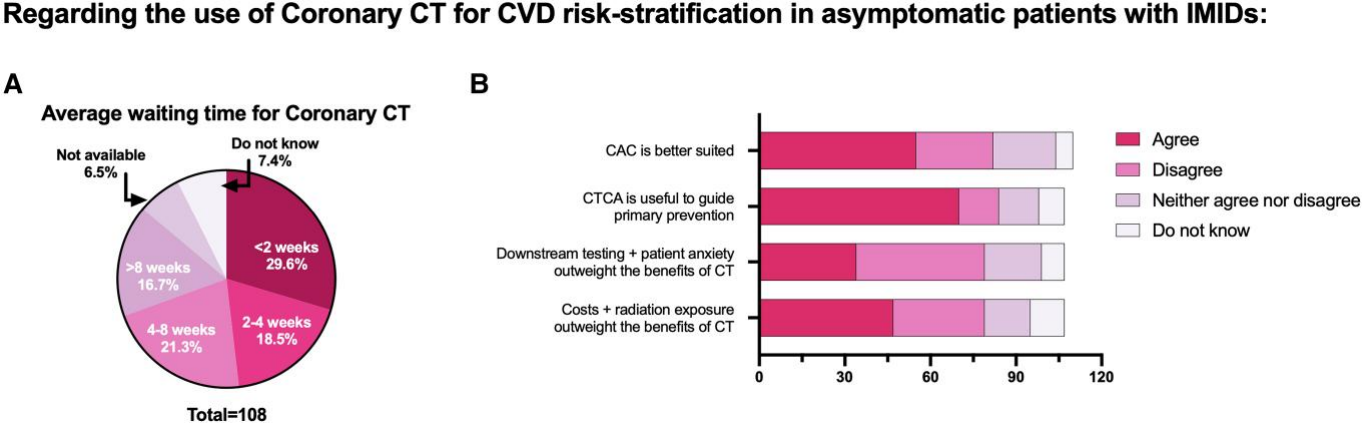
Use of coronary CT for CVD risk-stratification in IMIDs. Graphs showing the average waiting times for coronary CT (*A*) and the perceived advantages and disadvantages of the use of CT in this setting (*B*).

Coronary CT remains one of the most readily available cardiovascular imaging modalities and was accessible within 2 weeks in 32 (29.6%) participant centres and in 2–4 weeks to a further 20 (18.5%) centres in the EACVI survey. Only 7 (6.5%) responded saying they do not have access to coronary CT in their local hospital (*Figure 4A*). CCTA was generally perceived as a useful tool to guide primary prevention therapies in patients with a borderline clinical risk score category, over and above CAC scoring alone. Attitudes towards the potential drawbacks of the use of CT in the setting of primary prevention were mixed. Approximately one-third of people indicated that they neither agreed nor disagreed that the increased downstream testing, additional costs, and radiation exposure for patients outweighed the benefits of using CCTA for CVD risk-stratification in patients with IMIDs (*Figure 4B*), reflecting a clear evidence gap on this topic. These findings were corroborated by the BSCI/BSCCT survey (*Figure 2D*).

However, there was a clear steer from the 6 members of the UK Cardio-IMID Patient Advisory Group who were interviewed about their views on the use of CCTA for atherosclerotic CVD screening, against this approach. These patients living with chronic IMIDs voiced concern about the prospect of uncovering a new CVD diagnosis because of the additional anxiety this information would cause when they did not have cardiovascular symptoms, suggesting that the roll-out of a national CCTA screening programme would need to be accompanied by the development of new educational resources to support the patients wishing to be enrolled.

### Use of CMR to assess myo-pericardial involvement in IMIDs

**Figure 5:**
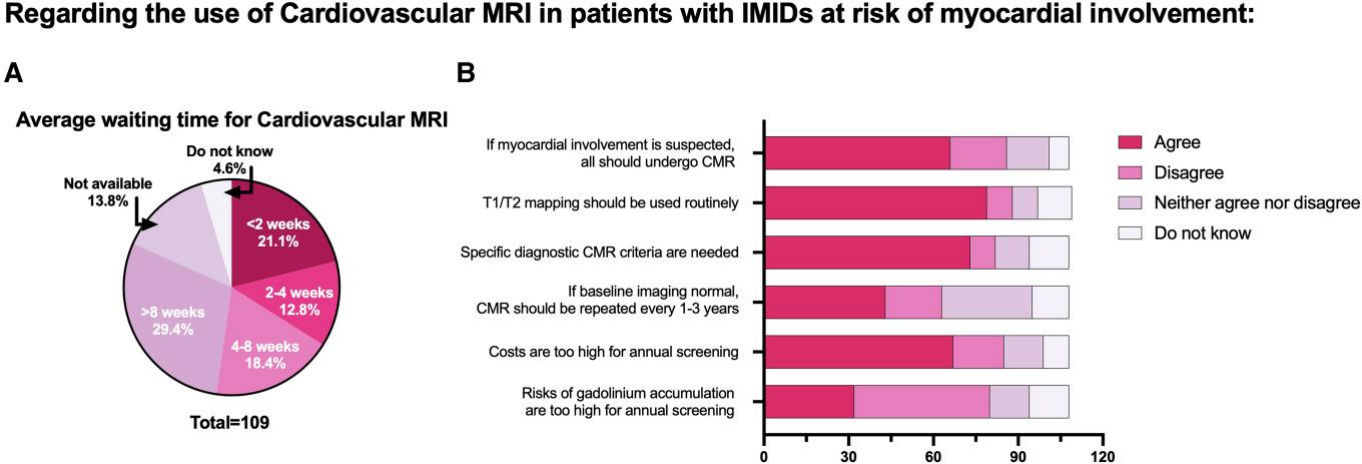
Use of CMR to assess myo-pericardial involvement in IMIDs. Graphs showing the average waiting times for CMR (*A*) and the perceived advantages and disadvantages of the use of CT in this setting (*B*).

Fewer responders in the EACVI survey had access to CMR compared to coronary CT, and 15 people (13.8%) could not access it locally as compared with 7 (6.5%) who could not access CT (*Figure 5A*). Average waiting times were overall longer, with 20 people (18.4%) waiting 4–8 weeks for CMR and 32 (29.4%) waiting longer than 8 weeks (*Figure 5A*). Despite these longer waiting times, there was consensus that CMR is the modality of choice if myocardial involvement in IMIDs is suspected. Moreover, there was strong support for the routine use of parametric mapping in this setting. However, the Lake Louise criteria developed for myocarditis were felt to inadequately capture the complexity of CMR findings in IMIDs, with as many as 73 participants (67.6%) advocating for new IMID-specific diagnostic CMR criteria in this high-risk population.^7^ The use of CMR for routine monitoring in IMIDs in the absence of symptoms was less widespread. While the potential risks of gadolinium contrast reactions and accumulation in tissues were not felt to be a significant barrier, the overall impression was that the cost of CMR was prohibitively high to justify annual screening in IMIDs. Interval CMR every 1–3 years in IMID patients at risk of myocardial involvement was felt to be a more acceptable option (*Figure 5B*).

### Use of PET imaging for disease diagnosis and monitoring in large vessel vasculitis

**Figure 6:**
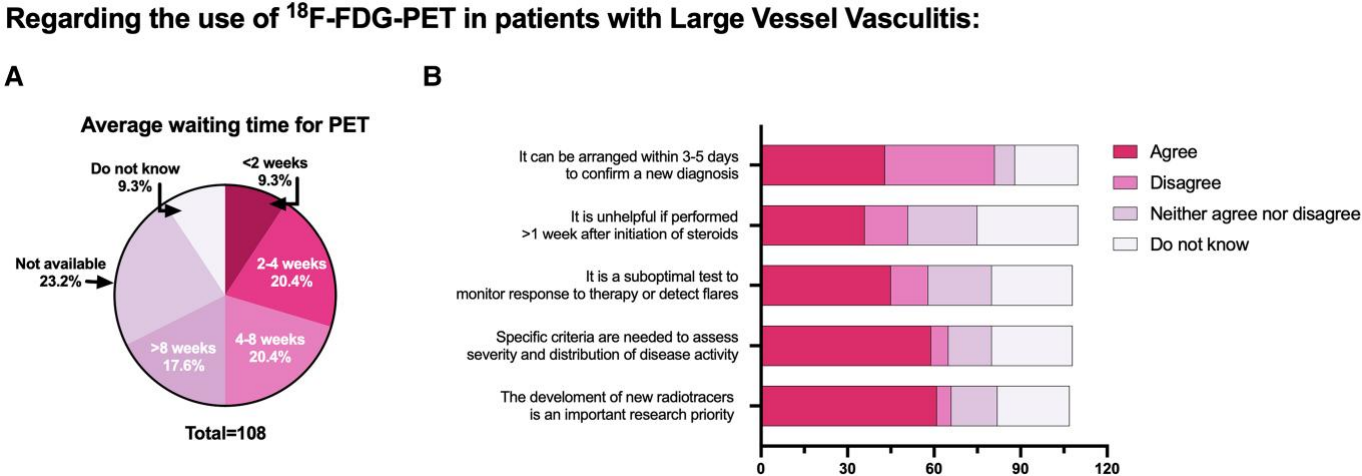
Use of PET imaging for disease diagnosis and monitoring in large vessel vasculitis. Graphs showing the average waiting times for ^18^F-FDG PET imaging (*A*) and perceived advantages and disadvantages of the use of PET imaging in this setting (*B*).

Direct access to ^18^F-fluorodeoxyglucose (FDG) positron emission tomography (PET) was more restricted than CT or CMR for those who took part in the survey, with 25 responders (23.2%) reporting no local availability. Where available, average waiting time for ^18^F-FDG PET varied widely and was <4 weeks in 32 centres (28.8%), 4–8 weeks in 22 (20.4%), and >8 weeks in 19 (17.6%) (*Figure 6A*). As a result, there was a clear divide between centres with access to PET within the guideline-recommended 3–5 days to confirm a diagnosis of large vessel vasculitis (LVV).^8^ Of those with access to PET imaging, 43 (39.1%) could achieve this target, and 38 could not (34.6%). A further 29 could not answer this question definitively, and neither agreed nor disagreed (*n* = 7, 6.4%), or did not know (*n* = 22, 20%) if they would be able to arrange a PET scan within 3–5 days to confirm a new diagnosis. There was a lack of consensus about the optimal timing for PET when used to diagnose LVV, as well as its potential role for disease monitoring (*Figure 6B*). Yet, 59 (54.6%) agreed that there is a need to improve specific imaging criteria or semi-quantitative uptake metrics used for assessing the severity and distribution of disease activity by PET imaging in LVV (*Figure 6B*).

### Clinical scenario about the use of imaging to guide CVD therapy in IMIDs

**Figure 7:**
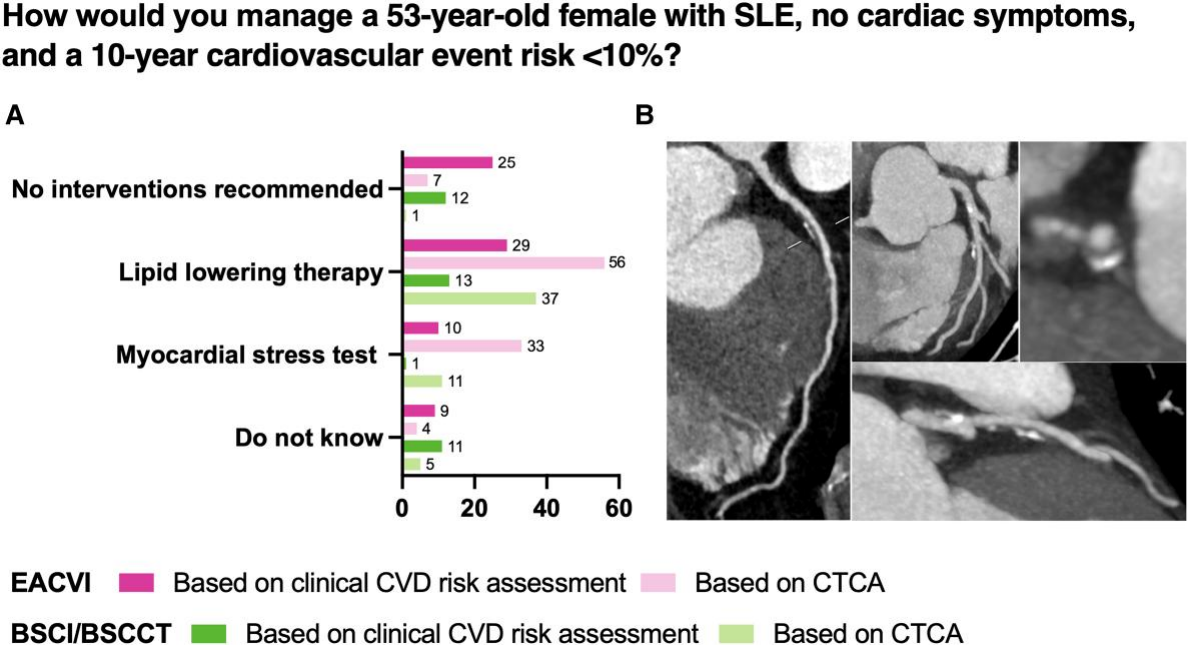
Responses to the clinical management scenario proposed to EACVI and BSCI/BSCCT members. Answers based on clinical CVD risk assessment with or without coronary CT findings (*A*). Representative CT showing moderate mid left anterior descending artery stenosis with positive vessel remodelling (*B*). SLE, systemic lupus erythematosus.

Both the EACVI and the BSCI/BSCCT e-surveys included a specific clinical management question framed around a clinical scenario of a 53-year-old woman with systemic lupus erythematosus (SLE) who had a 10-year risk of CVD events of 3.3% based on SCORE2, and 4.4% based on QRISK3. When asked about initiation of statin therapy for this individual with SLE, 29 participants (26.6%) from the EACVI survey opted for lipid-lowering therapy, 25 (22.9%) did not feel therapeutic intervention was indicated, and 36 (33%) chose to further investigate with CCTA (*Figure 7A*). Interestingly, when the data were analysed, separating EU vs. non-EU data, we found that 27 (29%) of our European responders were likely to prescribe lipid-lowering therapy based on CVD risk score alone, vs. only 2 (11.1%) of non-Europeans. Attitudes towards CCTA were consistent between the two groups. When presented with the results of the CCTA, which showed a CAC score of 7.7 (84th percentile for age and gender match) and moderate mid left anterior descending artery stenosis with positive vessel remodelling (*Figure 7B*), nearly twice as many people opted for lipid-lowering therapy and three times as many for myocardial perfusion imaging compared to the management that was proposed before being given CT results. Only four (3.4%) remained unsure of how to proceed. Similar results were observed when the same clinical scenario was presented to BSCI/BSCCT members (*Figure 7A* and *B*).

### Future research priorities

Forty-four (40.4%) responding to the EACVI survey felt that multi-modality imaging was the most promising direction for future research in the field, to allow comprehensive assessment of cardiovascular involvement in patients with IMIDs. Coronary CT remains the most widely available imaging modality, and the role of CCTA for CVD risk-stratification merits further investigation. Interestingly, 32 people (29.4%) saw an opportunity in combining imaging data with biomarkers and genetic profiling, reflecting a push towards the development of personalized risk profiles and treatment strategies. Finally, the survey highlighted that the development of novel imaging probes to visualize molecular markers of cardiovascular inflammation, fibrosis, and endothelial dysfunction is an important research priority, with 61 (57%) of responders agreeing that this topic should be a focus for future research and only 5 (4.7%) disagreeing (*Figure 6B*). Ultimately, this would result in a better understanding of CVD in IMIDs.

## Discussion

This EACVI survey investigated the current use of cardiovascular imaging in cardio-rheumatology across Europe and beyond, including specific uses of imaging for diagnosis and monitoring cardiovascular complications in patients with IMIDs. We found that across Europe, there was wide variation in access to advanced cardiovascular imaging modalities and local practices for the evaluation of cardiovascular complications in IMIDs, suggesting a need for standardized care pathways.

### A multidisciplinary approach to CVD risk-stratification

Guidelines from the European Alliance of Associations for Rheumatology (EULAR) recognize that patients with IMIDs carry an increased risk of cardiovascular disease and recommend more frequent screening than in the general population.^3,4^ However, clinical CVD risk scores developed for the general population have been shown to inaccurately predict risk in IMIDs.^5^ This finding is highlighted by our survey, with 104 responders (94.5%) advocating for bespoke CVD risk stratification strategies in patients with IMIDs. A recent survey in the United Kingdom found that the majority of rheumatologists discuss CVD risk with their patients, but as many as 46% do not feel confident interpreting CVD risk tests, including lipid profile, and 95% would defer initiation of risk-reduction strategies to primary care.^9^ On the other hand, we identified a clear gap in training opportunities in the field of cardio-rheumatology, limiting the expertise of cardiologists who may only sporadically manage patients with IMIDs. This highlights the importance of a multi-disciplinary approach in the care of these patients, involving both medical specialties as well as primary care physicians. While our survey found that a dedicated cardio-rheumatology service was only available in a minority of the centres interviewed, 92 participants (82.9%) thought that it would benefit patient care, lead to standardization of treatment and foster cross-disciplinary collaboration. Hence, the development of dedicated cardio-rheumatology services and more opportunities for training in this field emerged as important unmet clinical needs that warrant further attention.

### Coronary CT for early detection of atherosclerotic disease

Coronary CT is an attractive option for early detection of subclinical atherosclerotic CVD and may be a useful adjunct test in patients with IMIDs who have a borderline indication for statins. Most studies on this topic have focused on the use of CAC scoring in addition to clinical CVD risk scores, showing the potential to re-classify asymptomatic patients with IMIDs who would have otherwise fallen in moderate-risk categories.^10,11^ However, unlike CAC scoring alone, CCTA allows detection of non-calcified plaques that are common in patients with IMIDs, who include younger females who lack traditional CVD risk factors. Our survey found greater agreement with the use of CCTA for CVD risk-stratification as compared with CAC scoring alone. The SCOT-HEART 2 trial is underway to determine whether screening asymptomatic individuals with CVD risk factors using CCTA will improve cardiovascular death and non-fatal myocardial infarction compared with clinical risk scores alone.^12^ Similar data will be required in IMIDs. The results from our surveys indicate an appetite in the cardiovascular imaging community to use CCTA in this setting to ensure preventive therapies are prioritized in the patients who will most benefit. However, the potential benefits of this imaging approach would need to be accompanied by effective counselling to mitigate the concerns raised by our Patient Advisory Group about the possible negative effects of additional testing on the psychological well-being of people living with chronic diseases.

### Cardiovascular CMR for detection of primary myocardial involvement

CMR is becoming more widely accessible, and current European guidelines recommend this imaging modality first-line when myocardial involvement in IMIDs is suspected.^13^ Despite the ability to detect myocardial oedema and scarring/fibrosis with T2-weighted imaging, multi-parametric mapping, and late gadolinium enhancement CMR, the diagnosis of IMID myocarditis remains challenging.^14^ This was recognized by the cardiovascular imaging community taking part in our survey, who called for new specific diagnostic criteria beyond the revised Lake Louise criteria for detecting myocardial involvement in IMIDs. This need is supported by a study that observed high specificity, but low sensitivity when applying revised Lake Louise criteria for myocarditis in IMIDs.^15^ Our study also identified an evidence gap and need for future guidance about the role of CMR for screening and monitoring of asymptomatic individuals with IMIDs at risk of myocardial involvement. According to our survey, the cost of CMR is a barrier to widespread uptake of annual screening for high-risk individuals with IMIDs, who are managed on a case-by-case basis currently.^16^

### PET imaging for the assessment of vascular wall inflammation

^18^F-FDG PET is a first-line recommended test to investigate suspected LVV with extracranial involvement. EULAR guidelines advocate scanning as early as possible, and ideally within 7 days of treatment initiation, due to the rapidly declining diagnostic accuracy and attenuation of arterial ^18^F-FDG PET signal in LVV after 3 days of high-intensity steroid treatment.^8^ However, less than half of the centres that took part in our survey could access PET imaging within this short timeframe. Perhaps as a reflection of this, and due to the intrinsic limitations of the use of ^18^F-FDG imaging for the assessment of vascular inflammation,^17^ responders indicated the development of novel PET tracers as a main research priority in this field. Indeed, whilst undoubtedly a fundamental part of the diagnostic workup of LVV, the role of ^18^F-FDG PET imaging in therapy monitoring is less clear due to a high prevalence of positive scans in patients who have achieved clinical remission.^18^

### Future directions

This survey emphasizes the importance of a multimodal approach to CVD risk stratification in IMIDs, aimed at developing a personalized risk profile. Previous research has shown that the addition of an imaging biomarker as personalized risk component in CVD risk-stratification promotes healthier lifestyles and improves the acceptance rates of preventive therapies,^19,20^ suggesting that cardiovascular imaging may be a valuable tool for the primary prevention of CVD in high-risk populations. Two large-scale prospective observational studies, the EACVI-INFLAME and UK CARDIO-IMID Registry Study (NCT07077304 and NCT06478277, respectively) are underway and will inform the integration of imaging into routine clinical practice. Technological advancements, including photon-counting CT scanners and the use of novel solutions for plaque quantification, may also lead to new opportunities for research studies about coronary risk stratification in people with IMIDs. Similarly, the advent of ultrasensitive, high-resolution, large field-of-view PET scanners could reduce scanning time and radiation exposure, therefore enabling serial imaging. This, in conjunction with the development of more specific radionuclide tracers targeting inflammation, fibrosis and endothelial dysfunction, could increase the scope for PET imaging in IMIDs. Wider access to CMR and lower cost are expected to arise from wider use of artificial intelligence in image acquisition and analysis, and will be critical to meet its role as the first-line investigation in myocarditis and pericarditis in recent ESC guidelines. In the future, there will be a need for standardization, training, and multidisciplinary collaboration to apply valid clinical interpretation to these novel imaging biomarkers.

### Limitations

Both the EACVI and BSCI/BSCCT surveys were completed on a volunteer basis; therefore, there is a risk of selection bias. Most responders worked in tertiary centres and were weighted towards cardiovascular imagers, so the results may not be generalizable across all healthcare settings. Of note, a large percentage of IMID patients in Europe is currently cared for in the primary care setting, where CVD risk assessment is likely to take place. The views of general practitioners are not captured in this survey and should be the subject of future research. Although this was a Europe-wide survey, the sample size was modest which could reflect the specialist topic and emerging field. Some countries contributed a very small number of responders; hence the data provided on access to advanced cardiovascular imaging may not be generalizable to all European centres. The survey was predominantly focused on cross-sectional cardiac imaging. Future surveys should address the role of echocardiography for the screening of asymptomatic IMID patients. There is a need for dedicated surveys to capture wider patient views.

## Conclusion

The results of this EACVI survey highlight several unmet clinical needs in the care of patients with IMIDs, reflecting the challenges posed by CVD risk assessment and mitigation in this high-risk population. Multi-modality cardiovascular imaging already plays a key role in identifying patients who might benefit from CVD risk modification strategies and immunomodulatory therapy, as is reflected by current guidelines. However, the scope for the clinical use of imaging in IMIDs will likely increase further as bespoke diagnostic criteria are developed and new technological advances emerge. Further evidence is needed to guide the routine use of cardiovascular imaging and shape future clinical practice guidelines specific to patients with IMIDs.

## Supplementary Material

qyag094_Supplementary_Data

## Data Availability

The data underlying this article will be shared on reasonable request to the corresponding author.

## Lead author biography

Marta is a Clinical Research Fellow/PhD Student in Cardiology at the Victor Phillip Dahdaleh Heart & Lung Research Institute, University of Cambridge. Her research focuses on vascular inflammation imaging and high-risk atherosclerotic plaque biology. She is a member of the Trainee Committee at the British Society of Cardiovascular Imaging/British Society of Cardiac Computed Tomography.
