# Supplementary material for: EACVI survey on the use of multi-modality cardiovascular imaging in immune-mediated inflammatory diseases
Source: Eur Heart J Imaging Methods Pract. 2026 May 16;4(1):qyag094. doi: 10.1093/ehjimp/qyag094 (PMC13253017; doi:10.1093/ehjimp/qyag094)
Supplement: qyag094_Supplementary_Data [file qyag094_supplementary_data.docx]

# **BSCI survey on the Use of Cardiac CT for cardiovascular risk-stratification in Immune Mediated Inflammatory Diseases**

## Background/ rationale

People with immune-mediated inflammatory diseases (IMIDs) are known to be at increased risk of cardiovascular disease, which is not captured by current clinical risk assessment tools. The aims of this survey are to better understand how cardiac CT is currently being used in patients with IMIDs (e.g., rheumatoid arthritis, psoriasis, systemic lupus erythematosus, vasculitis) in the UK, and to identify the key priority areas for future research on this topic.

**Hospital setting**

1. What is your medical specialty? (Please select one option)
   1. Radiology
   2. Cardiology
   3. Rheumatology
   4. Renal medicine
   5. Internal medicine
   6. Nuclear medicine
   7. Other______
2. What is your current medical position? (Please select one option)
   1. Consultant / Associate Specialist
   2. Postgraduate Medical Trainee/ Specialty Fellow
   3. Research Fellow
   4. Other______
3. What type of hospital do you mostly work in? (Please select one option)
   1. Tertiary Centre/ University Hospital
   2. District general hospital
   3. Community healthcare
   4. Academic University
   5. Private hospital
   6. Other______
4. Which geographical area do you practice in? (Select one)
   1. England
   2. Scotland
   3. Wales
   4. Norther Ireland
   5. Other______
5. How often to do you see patients with IMIDs in your clinical practice? (Please select one option)
   1. Never
   2. Sometimes (every 3-6 months)
   3. Often (every month)
   4. Very often (every week)
   5. Do not know

**Assessment of cardiovascular risk in asymptomatic individuals with IMIDs**

1. In your opinion, is there a need for specific cardiovascular risk-stratification strategies for people with IMIDs in the absence of cardiac symptoms? (Please select one option)
   1. Yes
   2. No
   3. Maybe
   4. More evidence is needed to answer this question
   5. Do not know
2. In your practice, IMID patients without cardiac symptoms are stratified for their atherosclerotic CVD risk based on (select all that apply):
   1. Rheumatological diagnosis
   2. Immune profile
   3. Duration and nature of immunosuppressive therapy
   4. Clinical CVD risk scores only (e.g., QRISK, SCORE)
   5. Cardiac biomarkers (Troponin and/or BNP)
   6. Electrocardiography
   7. Abnormal findings on cardiac imaging
   8. Do not know
3. If you rely on abnormal findings on cardiac imaging, which modality are you most likely to use? (Please select one option)
   1. Transthoracic echocardiography
   2. Coronary Artery Calcification (CAC) scanning
   3. CT coronary Angiography (CTCA)
   4. Non-invasive stress or perfusion testing
   5. No imaging routinely performed in the absence of symptoms
   6. Do not know
   7. Other______
4. If CCTA were available as a screening tool, which additional factors would you consider when deciding which patients to refer?
   1. Disease duration
   2. Time spent in flare
   3. Immune profile
   4. Cumulative steroid dose
   5. Choice of biologic immunomodulators
   6. Traditional cardiovascular risk factors
   7. Other______
5. What challenges do you encounter when assessing CVD risk in IMIDs? (Please select all that apply)
   1. Lack of traditional CVD risk factors
   2. Failure of clinical CVD scores to capture disease-specific risk factors (e.g. rheumatological disease duration, time spent in flare, cumulative steroid dose)
   3. Radiation exposure limiting the use of imaging in IMID patients (who are often younger females that are likely to require multiple imaging tests throughout their disease)
   4. Absence of a designated Cardio-Rheumatologist Specialist or clear guidelines to advise on optimal CVD screening strategies and the use of preventative therapies in this population
   5. Barriers to accessing specialist cardiovascular imaging tests
   6. Lack of awareness of increased CVD risk in IMIDs because this topic is not well covered by current general cardiology or rheumatology training (and/or limited local opportunities to gain experience outside of training)
   7. Do not know
   8. Other______

**Use of CCTA in IMIDs**

Do you agree or disagree with the following statements regarding the use of CTCA for CVD risk-stratification in IMID patients with no cardiovascular symptoms:

1. CAC scoring would be better suited for asymptomatic screening than CTCA (Select one option)
   1. Agree
   2. Disagree
   3. Neither agree nor disagree
   4. Do not know
2. CTCA could be a useful to guide preventative therapies in asymptomatic patients with borderline clinical risk profiles
   1. Agree
   2. Disagree
   3. Neither agree nor disagree
   4. Do not know
3. CTCA is not helpful in asymptomatic individuals because it would lead to un-necessary downstream testing and increased patient anxiety
   1. Agree
   2. Disagree
   3. Neither agree nor disagree
   4. Do not know
4. The costs associated with CTCA outweigh the potential benefits for asymptomatic CVD screening in IMID patients (Select one option)
   1. Agree
   2. Disagree
   3. Neither agree nor disagree
   4. Do not know
5. The radiation exposure associated with CTCA outweigh the potential benefits for asymptomatic CVD screening in IMID patients (Select one option)
   1. Agree
   2. Disagree
   3. Neither agree nor disagree
   4. Do not know
6. What further evidence would be most helpful to determine the clinical role of CTCA for risk-stratification in IMIDs with no cardiovascular symptoms (Select one option):
   1. A clinical outcomes trial comparing CVD risk-stratification by CAC scoring vs. standard clinical risk scores alone
   2. A clinical outcomes trial comparing CVD risk-stratification by CCTA vs. standard clinical risk scores alone
   3. A clinical outcomes trial comparing CVD risk-stratification by CAC vs. CCTA
   4. A clinical outcomes trial comparing CVD risk-stratification by CCTA vs. myocardial perfusion/ stress-imaging
   5. Do not know
7. What other information is needed to inform the CTCA use in IMIDs with no cardiovascular symptoms
   1. Patient-centred outcome trial

(high priority/ moderate priority/ low priority)

- 1. Cost-effectiveness analysis

(high priority/ moderate priority/ low priority)

- 1. Disease-specific clinical efficacy data

(high priority/ moderate priority/ low priority)

**Clinical scenario**

1. A 53-year-old female has a history of systemic lupus erythematosus but no cardiac symptoms. Her QRISK3 taking into account her SLE and latest lipid profile including LDLc 3.4 mmol/L yields a 10-year cardiovascular event risk of 4.4%. How would you manage her CVD risk? (Select one option)
   1. The risk is <5% so no interventions are recommended
   2. Lipid-lowering therapy and other risk modifiers are indicated
   3. CAC and/or CTCA scanning is indicated for further risk-stratification
   4. Myocardial stress test (stress echo, stress perfusion CMR, nuclear stress scan) is indicated for further risk-stratification
   5. Do not know
2. A CT scan is requested. The CAC score is 7.7 (84^th^ percentile for age and gender match). There is a moderate mid left anterior descending artery plaque with positive vessel remodelling. How does this change your management? (Select one option)
   1. No change because the patient is asymptomatic and has a low CAC score
   2. Lipid lowering therapy and other risk modifying interventions are indicated
   3. Functional testing (stress echo, stress perfusion CMR, nuclear stress scan) to determine the haemodynamic significance of the lesion is indicated
   4. Invasive angiography ± pressure wire study is indicated
   5. Do not know

**Future directions**

1. What advancements or innovations in CT imaging do you believe hold the most promise for improving CVD risk stratification in patients with IMIDs? (Select one option)
   1. Quantitative plaque analysis
   2. Coronary inflammation assessment by perivascular adipose tissue density/ radiomics
   3. Photon counting CT
   4. Implementing AI algorithms for automated interpretation of CTCA scans
   5. Low radiation allowing repeat imaging to assess treatment response
   6. Do not know

# **EACVI Cardio-Rheumatology Questionnaire**

Aim: To scope the current landscape on Cardio-Rheumatology services

**Hospital setting and availability of Cardio-Rheumatology services:**

1. What country are you practising in?
   1. Automated drop-down menu all countries
2. What is your current medical position? (chose from drop down list)
   1. Consultant / Attending physician/ Associate Specialist
   2. Postgraduate Medical Trainee/ Medical Resident/ Specialty Fellow
   3. Research Fellow
   4. Other______
3. What type of hospital do you work in? (Please chose one option)
   1. Tertiary Centre/ University hospital
   2. Secondary Centre/ District hospital
   3. Private Hospital
   4. Other______
4. What is your medical speciality? (Please select one option)
   1. Cardiology
   2. Rheumatology
   3. Renal medicine
   4. Internal medicine
   5. Radiology
   6. Nuclear medicine
   7. Other______
5. Please select the services you have available locally (please chose all applicable options):
   1. Cardio-Rheumatology Clinic
   2. Cardio-Rheumatology Multidisciplinary Team (MDT) meeting
   3. A designated cardiologist who specialises in the management of patients with IMIDs
   4. A designated rheumatologist or nephrologist who specialises in the management of patients with IMIDs
   5. None of the above
   6. Do not know
   7. Other______
6. Do you think your hospital or clinic would benefit from a dedicated Cardio-Rheumatology service? (Please select one option)
   1. Yes, this is likely to improve patient care
   2. Yes, this may lead to the development of hospital policies to standardise the care of otherwise complex patients
   3. Yes, this would foster multidisciplinary collaboration between specialties
   4. No, the volume of IMID patients is small and wouldn’t justify a dedicated service
   5. No, these patients should remain primarily under the care of a tertiary centre
   6. Other______
7. Are there specific training opportunities in Cardio-Rheumatology available locally?
   1. Yes, in my hospital
   2. Yes, in my region
   3. Yes, in my country
   4. No
   5. Do not know
8. How often to do you see patients in your practice that could benefit from a Cardio-Rheumatology consultation? (Please select one option)
   1. Very often (every week), please specify the number per week
   2. Often (every month), please specify the number per month
   3. Sometimes (every few months)
   4. Rarely (a few times a year or less)
   5. Never
9. Which specialty is primarily responsible for requesting cardiac investigations other than transthoracic echocardiogram (e.g. Cardiovascular MRI, Coronary Computed Tomography Angiography) in patients with IMIDs in your institution?
   1. Cardiology
   2. Rheumatology
   3. Nephrology
   4. Internal medicine
   5. Variable (depends on which specialty the patient is under)
   6. Multidisciplinary Team (MDT)
10. Please select the availability and average waiting times for routine imaging in individuals with IMIDs in your institution (drop down menu: <2 weeks, <4 weeks, 4-8 week, >8 weeks, not available at all):
    1. Transthoracic echocardiography
    2. Coronary Computed Tomography Angiography (CCTA)
    3. Cardiovascular Magnetic Resonance Imaging (CMR)
    4. Nuclear myocardial perfusion imaging (SPECT)
    5. ^18^F-Fluorodeoxyglucose (FDG) Positron Emission Tomography (PET

**Assessment of cardiovascular risk in asymptomatic individuals with IMIDs**

1. In your practice, who who typically decides whether asymptomatic patients with IMIDs should be screened for Cardiovascular Disease and/or recommends preventative therapies?
   1. General practitioner
   2. Cardiologist
   3. Rheumatologist or Nephrologists
   4. Internal Medicine Physician
   5. Multidisciplinary Team - if Yes, please list which specialities involved (free text)
   6. No clear person responsible
2. In your opinion, is there a need for more bespoke CVD risk-stratification/ screening strategies for patients with IMIDs who do not have any cardiac symptoms?
   1. Yes, clinical CVD risk scores are known to underestimate risk in IMIDs
   2. Yes, clinical CVD risk scores fail to capture disease-specific risk factors (e.g. disease duration, time spent in flare, cumulative steroid dose)
   3. Yes, better guidance for preventative strategies is needed in IMIDs
   4. No, disease-specific clinical CVD risk score (e.g. the Expanded Risk in Rheumatoid Arthritis (ERS-RA)) do not outperform those used in the general population
   5. No, the burden of screening would outweigh possible benefits
   6. Other______
3. In your practice, IMID patients without cardiac symptoms are stratified for their atherosclerotic CVD risk on the basis of (select one or more):
   1. Rheumatological diagnosis
   2. Immune profile
   3. Duration and nature of immunosuppressive therapy
   4. Clinical CVD risk scores only (e.g., QRISK, SCORE, ASCVD)
   5. Cardiac biomarkers (Troponin and/or BNP)
   6. Electrocardiography
   7. Abnormal findings on cardiac imaging
   8. Do not know
4. What is your approach to the risk-stratification for atherosclerotic cardiovascular disease (CVD) in patients with IMIDs who do not have cardiac symptoms? (Please select all that apply):
   1. CT Coronary Artery Calcium (CAC) scoring
   2. Coronary CT angiography (CCTA)
   3. Stress imaging with echocardiography
   4. Stress perfusion imaging with Cardiovascular MRI (CMR)
   5. Stress perfusion imaging with Positron Emission Tomography (PET)
   6. Stress perfusion imaging with Single Photon Emission Tomography (SPECT)
   7. No imaging routinely performed in the absence of symptoms
   8. Other______
5. If designing a study/trial comparing long-term clinical outcomes using atherosclerotic CVD risk-stratification in IMID patients without cardiac symptoms, how would you prioritise the need for the following?
   1. Standard clinical risk scores alone vs. CAC scoring

(high priority/ moderate priority/ low priority/you do not feel qualified to answer)

- 1. Standard clinical risk scores alone vs. CCTA

(high priority/ moderate priority/ low priority/you do not feel qualified to answer)

- 1. Standard clinical risk scores alone vs. myocardial perfusion/ stress-imaging

(high priority/ moderate priority/ low priority/you do not feel qualified to answer)

- 1. Standard clinical risk scores alone vs. CCTA vs. myocardial perfusion/ stress-imaging

(high priority/ moderate priority/ low priority/you do not feel qualified to answer)

**Use of CCTA in IMIDs**

1. To what extent do you agree or disagree with the following statements regarding the use of CCTA for CVD risk-stratification in asymptomatic IMID patients:
   1. CAC scoring would be better suited for asymptomatic screening than CCTA

(agree/ disagree/ neither agree nor disagree/do not know)

- 1. CCTA could be a useful to guide preventative therapies in asymptomatic patients with borderline clinical risk profiles

(agree/ disagree/ neither agree nor disagree/do not know)

- 1. CCTA is not helpful in asymptomatic individuals because it would lead to un-necessary downstream testing and increased patient anxiety

(agree/ disagree/ neither agree nor disagree/do not know)

- 1. The costs and/or radiation exposure associated with repeated CCTA outweigh the potential benefits for asymptomatic CVD screening in IMID patients

(agree/ disagree/ neither agree nor disagree/do not know)

**Use of CMR in IMIDs**

1. To what extent do you agree or disagree with the following statements regarding the use of cardiovascular MRI (CMR) in IMIDs:
   1. All patients with IMID and suspected myocardial involvement should undergo CMR

(agree/ disagree/ neither agree nor disagree/do not know)

- 1. Parametric mapping (T1/T2 mapping) should be used routinely in patients with IMID and suspected myocardial involvement

(agree/ disagree/ neither agree nor disagree/do not know)

- 1. Specific diagnostic CMR criteria beyond the Lake Louise criteria for inflammatory myocarditis are needed

(agree/ disagree/ neither agree nor disagree/do not know)

- 1. For patients with IMIDs at risk of myocardial involvement, if the baseline imaging is normal, CMR should be repeated every 1 to 3 years?

(agree/ disagree/ neither agree nor disagree/do not know)

- 1. The costs of CMR of repeated CMR are too high for annual screening

(agree/ disagree/ neither agree nor disagree/do not know)

- 1. The potential risk of gadolinium accumulation in tissues are too high for annual screening

(agree/ disagree/ neither agree nor disagree/do not know)

**Use of PET in IMIDs**

1. To what extent do you agree or disagree with the following statements regarding the use of PET imaging in IMIDs:
   1. When required to confirm a new diagnosis of large vessel vasculitis (LVV), 18F-FDG PET can typically be arranged within 3-5 days in my hospital or regional referral centre

(agree/ disagree/ neither agree nor disagree/do not know)

- 1. In my experience, 18F-FDG PET is usually unhelpful if performed > 1 week of steroid initiation for LVV because of the vascular inflammatory signal is already suppressed

(agree/ disagree/ neither agree nor disagree/do not know)

- 1. In my experience, 18F-FDG PET imaging is an important initial diagnostic test for LVV, but repeated imaging is less useful for monitoring therapy response or detecting disease flares

(agree/ disagree/ neither agree nor disagree/do not know)

- 1. Specific imaging criteria or semi-quantitative uptake metrics are needed for assessing the severity and distribution of disease activity by PET imaging in LVV beyond visual scoring systems

(agree/ disagree/ neither agree nor disagree/do not know)

- 1. The development of new PET radiotracers for imaging vascular inflammation in LVV is an important research priority

(agree/ disagree/ neither agree nor disagree/do not know)

**Clinical scenario**

1. A 53-year-old female with systemic lupus erythematosus (SLE) but no cardiac symptoms has a 10-year cardiovascular event risk of 3.3% based on the Systematic Coronary Risk Evaluation (SCORE2) model. How would you manage her CVD risk?
   1. The risk is <5% so no interventions are recommend
   2. Lipid-lowering therapy and other risk modifiers are indicated
   3. CAC and/or CCTA scanning is indicated for further risk-stratification
   4. Myocardial stress test (stress echo, stress perfusion CMR, nuclear stress scan) is indicated for further risk-stratification
   5. I don’t know
2. A CT scan is requested. The CAC score is 7.7 (84th percentile for age and gender match). There is a moderate mid left anterior descending artery plaque with positive vessel remodelling. How does this change your management?
   1. No change because the patient is asymptomatic and has a low CAC score
   2. Lipid lowering therapy and other risk modifying interventions are indicated
   3. Functional testing (stress echo, stress perfusion CMR, nuclear stress scan) to determine the haemodynamic significance of the lesion is indicated
   4. Invasive angiography ± pressure wire study is indicated
   5. I don’t know
3. What advancement or innovation in cardiovascular imaging is the most promising for improving the care of patients with IMIDs? (Select one option)
   1. Combining different imaging modalities to provide a comprehensive assessment of cardiovascular involvement
   2. Utilizing speckle tracking echocardiography or feature tracking MRI to assess myocardial deformation and identify subtle changes
   3. Implementing AI algorithms for automated interpretation of cardiovascular imaging studies
   4. Developing novel imaging probes to visualize molecular markers of cardiovascular inflammation, fibrosis, and endothelial dysfunction
   5. Integrating handheld ultrasound devices into rheumatology clinics for real-time assessment of cardiac function and detection of pericardial effusions or valvular abnormalities
   6. Integrating imaging data with other diagnostic modalities such as laboratory tests or genetic markers to create personalized risk profiles and treatment strategies
   7. Other______
